# Endothelial Nitric Oxide Pathways in the Pathophysiology of Dengue: A Prospective Observational Study

**DOI:** 10.1093/cid/cix567

**Published:** 2017-06-29

**Authors:** Sophie Yacoub, Phung Khanh Lam, Trieu Trung Huynh, Hong Hanh Nguyen Ho, Hoai Tam Dong Thi, Nguyen Thu Van, Le Thi Lien, Quyen Nguyen Than Ha, Duyen Huynh Thi Le, Juthathip Mongkolspaya, Abigail Culshaw, Tsin Wen Yeo, Heiman Wertheim, Cameron Simmons, Gavin Screaton, Bridget Wills

**Affiliations:** 1 Oxford University Clinical Research Unit, Wellcome Trust Major Overseas Programme, Hanoi and Ho Chi Minh City, Vietnam;; 2 Department of Medicine, Imperial College London, United Kingdom;; 3 Hospital for Tropical Diseases, Ho Chi Minh City, and; 4 National Hospital for Tropical Diseases, Hanoi, Vietnam;; 5 Menzies School of Health Research, Darwin, Australia;; 6 Lee Kong Chian School of Medicine, Nanyang Technological University, Singapore;; 7 Nuffield Department of Medicine, University of Oxford, United Kingdom; and; 8 Department of Microbiology and Immunology, University of Melbourne, Australia

**Keywords:** dengue, endothelial function, nitric oxide, l-arginine, arginase

## Abstract

**Background:**

Dengue can cause increased vascular permeability that may lead to hypovolemic shock. Endothelial dysfunction may underlie this; however, the association of endothelial nitric oxide (NO) pathways with disease severity is unknown.

**Methods:**

We performed a prospective observational study in 2 Vietnamese hospitals, assessing patients presenting early (<72 hours of fever) and patients hospitalized with warning signs or severe dengue. The reactive hyperemic index (RHI), which measures endothelium-dependent vasodilation and is a surrogate marker of endothelial function and NO bioavailability, was evaluated using peripheral artery tonometry (EndoPAT), and plasma levels of l-arginine, arginase-1, and asymmetric dimethylarginine were measured at serial time-points. The main outcome of interest was plasma leakage severity.

**Results:**

Three hundred fourteen patients were enrolled; median age of the participants was 21(interquartile range, 13–30) years. No difference was found in the endothelial parameters between dengue and other febrile illness. Considering dengue patients, the RHI was significantly lower for patients with severe plasma leakage compared to those with no leakage (1.46 vs 2.00; *P* < .001), over acute time-points, apparent already in the early febrile phase (1.29 vs 1.75; *P* = .012). RHI correlated negatively with arginase-1 and positively with l-arginine (*P* = .001).

**Conclusions:**

Endothelial dysfunction/NO bioavailability is associated with worse plasma leakage, occurs early in dengue illness and correlates with hypoargininemia and high arginase-1 levels.

## Teaser

Endothelial dysfunction/reduced nitric oxide bioavailability is associated with worse plasma leakage in dengue patients and occurs early in the course of the disease. Endothelial dysfunction correlates with lower plasma l-arginine and higher arginase-1 levels.

Dengue continues to cause substantial global morbidity, with around 100 million clinically apparent cases estimated to occur every year [1]. Although dengue can present with a broad spectrum of clinical phenotypes, the defining feature of severe disease is altered vascular permeability resulting in a unique plasma leakage syndrome, which can progress to hypovolemic shock, known as dengue shock syndrome (DSS). Typically DSS occurs during a critical phase around day 4–6 of illness as fever resolves [2]. The mechanisms underlying the increased vascular permeability remain to be defined, but endothelial activation/dysfunction is thought to play a key role [3]. Investigating endothelial function clinically is difficult, but novel devices that measure endothelial-dependent vasodilation have been developed over the last decade, which involves physiological stimulation of endothelial release of nitric oxide (NO), reflecting local NO bioavailability [4]. Endothelial dysfunction is a systemic process that results in reduced NO release, causing impaired endothelial-dependent vascular relaxation, which can be quantified peripherally [5].

Alterations in a variety of biochemical precursor molecules and enzymes may result in impaired release of endothelial-derived NO, including deficiency of the substrate l-arginine in the vascular endothelium, inhibition of the enzyme endothelial NO synthase (eNOS), and deficiency of eNOS enzymatic cofactors. Other mechanisms that may alter vascular NO responses include competitive inhibition of NOS by endogenous methylarginines, particularly asymmetric dimethylarginine (ADMA), and increased expression or activity of the enzyme arginase-1 that metabolizes l-arginine [6, 7]. Derangements in these pathways have been found to contribute to endothelial dysfunction associated with the vascular complications observed in sepsis and malaria [8–10].

Although endothelial activation is known to occur in dengue, evidenced by increased release of various adhesion molecules [11, 12], very few data describing dysfunction of the endothelial NO–l-arginine pathway exist, and associations with dengue disease severity remain unknown.

Describing endothelial function across the spectrum of disease and exploring changes during the early febrile phase could identify potentially useful markers for subsequent development of shock, as well as providing insight into the underlying pathogenesis. We therefore set out to investigate associations between endothelial function (reactive hyperemic index [RHI]), plasma levels of l-arginine, ADMA, and arginase-1, in patients with dengue compared with other febrile illness (OFI) at serial time-points during the evolution of the acute illness. The main outcome of interest was plasma leakage severity, with a secondary outcome of mucosal bleeding. We hypothesized (1) that patients with dengue have worse endothelial dysfunction compared to patients with OFI, and (2) that, in the dengue group, endothelial dysfunction is associated with more severe plasma leakage. We also wished to explore associations between key molecules in the NO pathway, endothelial dysfunction, and clinical outcomes.

## METHODS

We performed a prospective observational study at the Hospital for Tropical Diseases (HTD), Ho Chi Minh City and the National Hospital for Tropical Diseases (NHTD), Hanoi, Vietnam, between June 2013 and October 2015. Ethical approvals were obtained from the Oxford Tropical Research Ethics Committee and at HTD and NHTD. Written informed consent was obtained from all participants or the parent/guardian of children [13]. Adults and children >3 years of age with a clinical diagnosis of possible dengue were eligible for enrollment into 1 of 2 study arms. In the outpatient arm, participants presenting within 72 hours of fever onset could be enrolled if no obvious alternative cause for the fever was apparent clinically [14]. For the inpatient arm, any individual admitted to NHTD or HTD with suspected dengue with warning signs or with severe dengue was eligible [15]. All patients were reviewed daily until fully recovered or for up to 6 days from enrollment, and also at a follow-up visit 14 days later.

### Clinical Endothelial Function Testing

Peripheral artery tonometry (EndoPAT, Itamar Medical, Israel) was used to measure digital vasodilator function. Digital pulse volume changes during reactive hyperemia were recorded in response to a 5-minute arterial occlusion, using a blood pressure (BP) cuff as per standardized methods [5]. A 5-minute baseline recording was performed, after which the BP cuff was rapidly inflated to a predetermined level (50 mm Hg above systolic BP, minimum of 200 mm Hg in adults and 50 mm Hg above systolic BP, no minimum for children) for 5 minutes. A further postocclusion phase was timed for another 5 minutes. The EndoPAT software calculates the RHI, which is the post- to preocclusion amplitude ratio, normalized to values obtained contemporaneously in the control arm. An abnormal ratio is defined by the manufacturer as RHI <1.67 [16]. EndoPAT was performed at 3 time-points; enrollment, defervescence/hospital discharge, and follow-up.

### Laboratory Parameters

A full blood count was performed daily, whereas liver and renal function was tested at enrollment and then subsequently if clinically indicated. A research plasma sample was stored every other day. Subsequently, a random selection of patients from each site and enrollment arm who had had EndoPAT tests performed had the following parameters measured at the same time-points specified above on the stored plasma, using commercial enzyme-linked immunosorbent assay (ELISA) kits for l-arginine and ADMA (DLD Diagnostika, Hamburg, Germany) and arginase-1 (Hycult Biotech, Uden, Netherlands).

### Dengue Diagnostics

An NS1 test (Platelia ELISA, Bio-Rad) and commercial immunoglobulin M (IgM) and immunoglobulin G (IgG) serology assays (Capture ELISA, Panbio, Australia) were used on acute and convalescent plasma. In addition, reverse-transcription polymerase chain reaction (RT-PCR) was performed on enrollment samples to identify the viral serotype and measure plasma viremia [17]. Patients were defined as having laboratory-confirmed dengue if the RT-PCR or NS1 was positive, and probable dengue if the IgM assay was positive at enrollment or if there was IgM seroconversion between paired specimens. A diagnosis of OFI was assigned to participants with no laboratory evidence of acute or recent flavivirus—that is, if they were negative for RT-PCR, NS1, and IgM/IgG on paired serology. Patients with negative tests at enrollment, but for whom convalescent plasma was not available, were considered unclassifiable.

### Clinical Endpoint Definitions

The primary endpoint was plasma leakage severity. Percentage hemoconcentration was defined as the ΔHCT (peak – baseline hematocrit / baseline hematocrit) × 100, and required at least 3 hematocrit recordings during the acute illness. The peak value was the highest measurement during the critical period (illness day 4–6), while the baseline hematocrit was the lowest value (in the following order) from the follow-up sample, a sample obtained within 72 hours of fever onset, or (for hospitalized patients only) the discharge sample, provided no parenteral fluid was administered during the preceding 12 hours. Plasma leakage severity was then graded into 3 categories: 0 indicates no clinically significant leakage (ΔHCT <15% and no signs of clinical fluid accumulation); 1 indicates moderate leakage (ΔHCT 15%–20% and/or any sign of clinical fluid accumulation); 2 indicates severe leakage (ΔHCT >20% and/or shock, or pleural effusion with respiratory compromise). Other outcomes assessed included the occurrence of mucosal bleeding, and overall dengue severity according to the World Health Organization (WHO) 2009 classification.

### Statistical Analysis

Data are presented as frequency (percentage) for categorical variables and median (interquartile range [IQR]) for continuous parameters. Comparisons of endothelial parameters were performed between confirmed dengue and OFI patients in the outpatient arm, based on logistic regression models with dengue diagnosis as the outcome of interest, and the endothelial parameters as covariates. Among patients with confirmed dengue, associations with plasma leakage and mucosal bleeding were then explored. These analyses, including trend analysis of endothelial parameters between the 3 plasma leakage grades, were based on linear regression models with endothelial parameters as covariates, and plasma leakage and mucosal bleeding as the outcomes. Overall comparisons (excluding measurements at follow-up) and comparisons in each illness phase were adjusted for age, sex, illness day at enrollment, and illness day of measurement, with additional adjustment for hospitalization in the analyses of clinical outcome. Generalized estimating equations were used to take into account multiple measurements per patient. Associations between endothelial parameters in patients with dengue were assessed by partial correlations controlling for potential confounding variables including age, sex, and illness day of measurement. The significance of partial correlations was assessed based on their Fisher transformation and corresponding bootstrap standard errors. To informally adjust for multiplicity, a significance level of .01 was used for all comparisons. All analyses were performed with the statistical software R version 3.2.2 and the companion package geepack version 1.2-0.

## RESULTS

**Figure 1.**
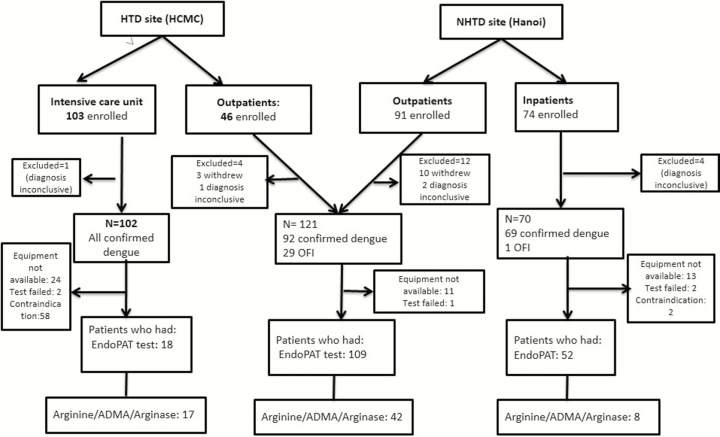
Study flow chart. Contraindications included age <10 years at the Hospital for Tropical Diseases site, platelet count <20 × 10^9^/L, or clinically unstable as decided by attending clinician. A random selection of patients who had peripheral artery tonometry testing were chosen to have plasma l-arginine, asymmetric dimethylarginine, and arginase tests. Abbreviations: ADMA, asymmetric dimethylarginine; EndoPAT, peripheral artery tonometry; HCMC, Ho Chi Minh City; HTD, Hospital for Tropical Diseases; NHTD, National Hospital for Tropical Diseases; OFI, other febrile illness.

In total, 314 patients were enrolled (Figure 1), of whom 21 withdrew or had inconclusive dengue diagnostics, leaving 293 for the final analysis. Overall, the median age of the participants was 21 (IQR, 13–30) years. The proportion of children (age <15 years) enrolled was 103 of 293 (35%); a higher proportion of children than adults was recruited in the intensive care unit (ICU): 86 of 102 (84%). There was an equal male-to-female ratio. Three patients had type 2 diabetes, 2 patients had controlled hypertension, and 6 patients smoked; the low frequency of these conditions were presumed not to be confounding on endothelial function as measured in this study. Of the 287 patients who had dengue virus (DENV) PCR performed, 201 (70%) were positive, with the following serotypes: 94 (47%) DENV-1; 28 (14%) DENV-2; 37 (18%) DENV-3; 41 (20%) DENV-4; and 1 (1%) mixed infection with DENV-1 and -4 (Table 1).

**Table 1.** Enrollment Characteristics for All Patients at Both Sites

Characteristic	No.	All Patients (N = 293)	No.	Outpatients (n = 121)	No.	Inpatients (n = 70)	No.	ICU (n = 102)
Age, y	293	21 (13–30)	121	26 (20–32)	70	28 (21–36)	102	11 (8–14)
Children (<15 y)	293	103 (35%)	121	12 (10%)	70	5 (7%)	102	86 (84%)
Male sex	293	155 (53%)	121	65 (54%)	70	36 (51%)	102	54 (53%)
Illness day	293	4 (3–6)	121	3 (2–3)	70	5 (4–6)	102	6 (5–6)
Dengue	293	263 (90%)	121	92 (76%)	70	69 (99%)	102	102 (100%)
OFI	293	30 (10%)	121	29 (24%)	70	1 (1%)	102	0 (0%)
PCR positive	287	201 (70%)	121	85 (70%)	68	49 (72%)	98	67 (68%)
Platelets, 10^9^/L	284	86 (30–153)	118	158 (129–198)	67	58 (31–108)	99	29 (19–42)
WBC, 10^9^/L	284	4.4 (3.0–6.1)	118	5.0 (3.3–7.2)	67	3.4 (2.5–4.7)	99	4.6 (3.0–5.8)
ALB, g/L	235	40.8 (34.2–46.0)	113	46.0 (42.0–48.1)	24	39.0 (36.0–45.0)	98	33.5 (29.2–37.3)
AST, U/L	249	60 (32–138)	117	32 (23–45)	40	62 (33–117)	92	164 (105–344)
Outcome variables								
Plasma leakage (yes)	245	126 (51%)	90	17 (19%)	63	21 (33%)	92	88 (96%)
Mucosal bleed (yes)	263	71 (27%)	92	26 (28%)	69	39 (57%)	102	6 (6%)
Hospitalized	263	199 (76%)	92	28 (30%)	69	69 (100%)	102	102 (100%)
WHO severity	263		92		69		102	
Dengue		79 (30%)		60 (65%)		19 (28%)		0 (0%)
WS		96 (37%)		30 (33%)		45 (65%)		21 (21%)
Severe		88 (33%)		2 (2%)		5 (7%)		81 (79%)

Outcome variables are for dengue-confirmed patients only. Summary statistics are absolute count (%) for categorical variables and median (interquartile range) for continuous data.

Abbreviations: ALB, albumin; AST, aspartate aminotransferase; ICU, intensive care unit; OFI, other febrile illness; PCR, polymerase chain reaction; WBC, white blood cell count; WHO, World Health Organization; WS, warning signs.

In patients with confirmed dengue, 126 of 245 (51%) developed the primary clinical endpoint of plasma leakage (both grades) and 71 of 263 (27%) developed mucosal bleeding (Table 1), while 199 of 263 (71%) were hospitalized at some point during their illness. Considering the WHO classification, there were roughly equal proportions of dengue patients with and without warning signs and with severe dengue (Table 1).

### Association of Endothelial Function, l-Arginine, Asymmetric Dimethylarginine, and Arginase-1 Between Dengue and Other Febrile Illness

There was no difference in the RHI or in plasma levels of l-arginine, ADMA, and arginase-1 between patients with dengue and OFI at any of the time-points (Table 2). Considering early illness (days 1–3) compared to follow-up (day >13), dengue patients had lower l-arginine levels (*P* < .001) (Supplementary Figure 1*A*) and l-arginine-to-ADMA ratio (*P* < .001), and higher arginase-1 levels (*P* = .004) (Supplementary Figure 2*A*). In the OFI group only, l-arginine was lower during days 1–3 compared to follow-up (49.1 vs 85.9 ng/mL; *P* < .001).

**Table 2.** Endothelial Function and Plasma l-Arginine, Arginase-1, and Asymmetric Dimethylarginine Levels Between Dengue and Other Febrile Illness

Time-point			OFI (n = 29)			Dengue (n = 92)			
	no.	No.	Median (IQR)	no.	No.	Median (IQR)	OR	(95% CI)	*P* Value
**Arginase, ng/mL**	**13**	**25**	**101.8 (63.4–203.1**)	**29**	**58**	**117.1 (70.8–213.8**)	**1.00**	**(1.00–1.01)**	**.344**
Days 1–3	12	12	122.6 (85.4–214.7)	24	25	183.3(112.0–221.2)	1.00	(1.00–1.01)	.318
Days 4–6	10	10	102.4 (55.4–196.5)	24	24	98.0 (48.6–170.8)	1.00	(1.00–1.01)	.527
Days 7–13	3	3	36.4 (35.4–69.1)	9	9	107.7 (92.4–122.1)	1.04	(.99–1.08)	.105
Day >13	8	8	78.0 (46.1–99.0)	16	16	91.6 (67.5–130.4)	1.00	(.99–1.02)	.706
**l** **-arginine, ng/mL**	**13**	**25**	**56.2 (43.9–64.0**)	**29**	**58**	**50.9 (38.0–67.0**)	**1.00**	**(.97–1.02)**	**.825**
Days 1–3	12	12	49.1 (42.8–58.9)	24	25	43.7 (36.9–62.0)	0.99	(.95–1.04)	.698
Days 4–6	10	10	60.1 (55.7–63.2)	24	24	51.9 (40.3–66.8)	0.99	(.97–1.02)	.549
Days 7–13	3	3	64.7 (53.5–67.1)	9	9	62.0 (55.2–69.0)	1.01	(.98–1.05)	.456
Day >13	8	8	85.2 (75.8–89.6)	16	16	77.0 (64.1–91.9)	1.01	(.97–1.04)	.754
**RHI**	**24**	**46**	**1.75 (1.46–2.44**)	**85**	**180**	**1.83 (1.53–2.29**)	**0.72**	**(.30–1.72)**	**.461**
Days 1–3	21	21	1.63 (1.42–2.47)	67	69	1.62 (1.43–2.02)	0.33	(.11–1.04)	.058
Days 4–6	20	21	1.74 (1.49–2.34)	65	70	1.94 (1.57–2.39)	1.15	(.40–3.33)	.794
Days 7–13	4	4	2.16 (1.92–2.27)	41	41	2.12 (1.79–2.56)	1.91	(.37–9.74)	.436
Day >13	17	17	2.00 (1.80–2.65)	45	45	1.86 (1.53–2.16)	0.35	(.11–1.14)	.082

All analyses were based on logistic regression with generalized estimation equation. Rows in bold represent the overall comparison including measurements from days 1 to 13. The analysis was adjusted for age, sex, illness day at enrollment, and illness day of measurement.

Abbreviations: CI, confidence interval; IQR, interquartile range; no., number of participants, No., number of measurements; OFI, other febrile illness; RHI, reactive hyperemic index.

### Associations of Endothelial Function, l-Arginine, Asymmetric Dimethylarginine, and Arginase-1 With Plasma Leakage Severity and Mucosal Bleeding in Dengue Patients

**Figure 2.**
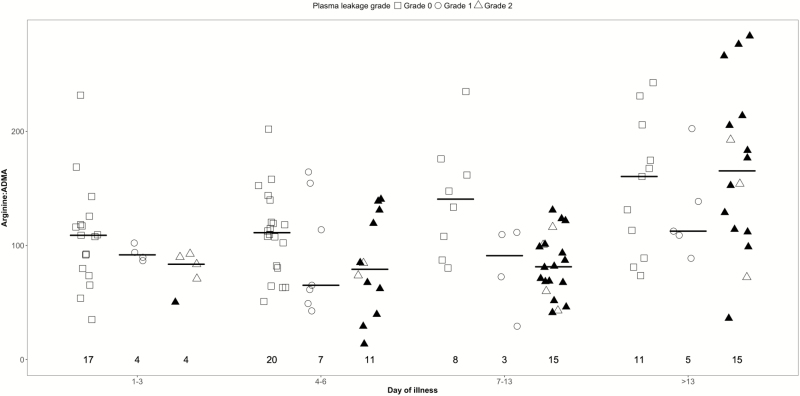
Scatterplot of l-arginine-to-asymmetric dimethylarginine (ADMA) ratios for dengue patients by plasma leakage severity and illness phase. Short gray line represents the median value of l-arginine-to-ADMA ratio for each illness phase. The number represents the number of patients that contributed to each group. These graphs are based on 54 patients with at least 1 measurement.

**Figure 3.**
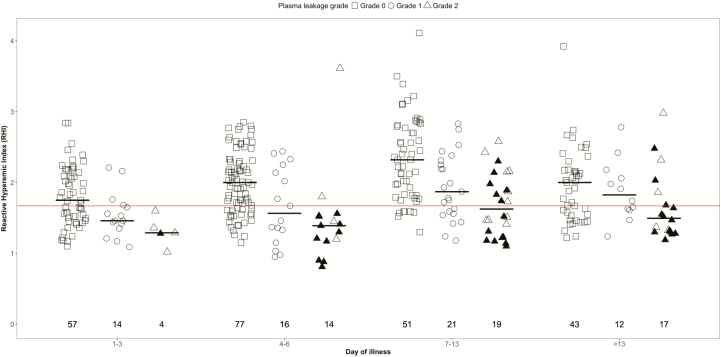
Scatterplot of endothelial function in dengue patients by plasma leakage severity and illness phase. Short gray line represents the median value of reactive hyperemic index (RHI) during each illness phase. The number represents the number of patients that contributed to each group. Redline represents the 1.67 cutoff, below which is defined as endothelial dysfunction. This graph is based on 109 patients with at least 1 measurement.

We observed no difference in plasma l-arginine, ADMA, and arginase-1 levels between the plasma leakage severity grades (Table 3 and Supplementary Table1*A*). There was a lower l-arginine-to-ADMA ratio for both leakage grades 1 and 2 vs no leakage during early convalescence (days 7–13) (Figure 2). The RHI was significantly lower for patients with grade 1 and grade 2 leakage compared to those with no leakage (days 1–13) (Table 3 and Figure 3). The RHI during the critical phase (days 4–6) and early convalescent phase (days 7–13) was significantly lower in those with plasma leakage grade 2 compared to no leakage. Using a linear regression trend test, there was a significant trend in the RHI between the plasma leakage grades overall (*P* < .001) and for days 1–3 (*P* = .001), days 4–6 (*P* = .002), and 7–13 (*P* < .001). There was no association between endothelial function, ADMA, or arginase-1 levels between patients with and without mucosal bleeding at any of the time points (Supplementary Table 2*A*).

**Table 3.** Association Between Endothelial Function, l-Arginine, Arginase-1, and Plasma Leakage by Illness Phase

Time-point	no.	No.	No Plasma Leakage (Grade 0)			Plasma Leakage Grade 1			Plasma Leakage Grade 2		Grade 1 vs 0			Grade 2 vs 0	
			Median (IQR)	no.	No.	Median (IQR)	no.	No.	Median (IQR)	Effect	(95% CI)	*P* Value	Effect	(95% CI)	*P* Value
**Arginase, ng/mL**	**23**	**45**	**109.7 (66.9–184.4**)	**8**	**15**	**117.5 (82.1–248.7**)	**19**	**37**	**116.3 (80.8–166.9**)	**50.87**	**(–62.63, 164.37)**	**.380**	**24.26**	**(–56.38, 104.90)**	**.556**
Days 1–3	17	17	136.3 (103.4–221.2)	4	4	158.6 (104.4–224.2)	4	5	183.4 (156.6–195.2)	–16.75	(**–**116.98, 83.49)	.743	**–**16.78	(**–**135.68, 102.13)	.782
Days 4–6	20	20	92.0 (54.8–121.2)	7	7	116.8 (52.9–237.8)	11	12	109.4 (84.9–154.7)	102.1	(**–**67.05, 271.10)	.237	105.67	(**–**26.61, 237.95)	.117
Days 7–13	8	8	113.0 (85.0–149.7)	3	4	121.2 (107.3–213.8)	15	20	105.9 (62.4–152.2)	90.48	(6.05–174.90)	.036	33.05	(**–**28.08, 94.19)	.289
Day >13	11	11	90.6 (57.3–115.4)	5	5	19.5 (18.0–75.2)	16	17	46.7 (36.0–79.3)	11.42	(**–**76.83, 99.67)	.800	18.36	(**–**27.83, 64.55)	.436
**l** **-arginine, ng/mL**	**23**	**45**	**60.0 (43.3–69.0**)	**8**	**15**	**47.6 (33.7–76.9**)	**19**	**37**	**42.8 (33.5–54.9**)	**–2.96**	**(–21.06, 15.15)**	**.749**	**–15.79**	**(–32.22, .65)**	**.060**
Days 1–3	17	17	47.2 (39.9–62.3)	4	4	51.7 (46.2–61.4)	4	5	33.5 (29.3–35.2)	11.65	(**–**2.28, 25.59)	.101	**–**3.87	(**–**23.64, 15.90)	.701
Days 4–6	20	20	61.4 (42.3–66.7)	7	7	34.3 (28.2–84.0)	11	12	42.1 (32.7–48.5)	**–**4.57	(**–**31.00, 21.86)	.735	**–**15.22	(**–**37.92, 7.48)	.189
Days 7–13	8	8	68.3 (60.2–92.5)	3	4	56.0 (31.7–83.2)	15	20	51.7 (36.2–66.2)	**–**9.74	(**–**37.58, 18.09)	.493	**–**21.07	(**–**46.55, 4.42)	.105
Day >13	11	11	83.3 (68.3–93.2)	5	5	70.5 (68.1–138.2)	16	17	95.9 (74.1–142.1)	3.58	(**–**26.63, 33.79)	.816	**–**4.23	(**–**33.23, 24.78)	.775
**RHI**	**101**	**194**	**2.00 (1.62–2.35**)	**25**	**54**	**1.66 (1.42–2.18**)	**22**	**44**	**1.46 (1.22–1.81**)	**–0.35**	**(–.50, –.20)**	**<.001**	**–0.53**	**(–.73, –.33)**	**<.001**
Days 1–3	57	57	1.75 (1.49–2.14)	14	15	1.46 (1.38–1.66)	4	5	1.29 (1.28–1.36)	**–**0.24	**–**.40, **–**.08)	.003	**–**0.34	(**–**.60, **–**.07)	.012
Days 4–6	77	82	2.00 (1.65–2.30)	16	16	1.56 (1.29–2.17)	14	15	1.39 (1.18–1.52)	**–**0.31	(**–**.59, **–**.03)	.032	**–**0.49	(**–**.84, **–**.13)	.007
Days 7–13	51	55	2.32 (1.83–2.80)	21	23	1.87 (1.58–2.28)	19	24	1.62 (1.29–2.02)	**–**0.46	(**–**.70, **–**.21)	<.001	**–**0.54	(**–**.77, **–**.30)	<.001
Day >13	43	43	2.00 (1.54–2.17)	12	12	1.82 (1.62–2.09)	17	18	1.50 (1.31–1.81)	**–**0.10	(**–**.30, .11)	.371	**–**0.25	(**–**.52, .02)	.066

For each variable, the first row corresponds to the overall comparison, which included all values except values on day >13, and were adjusted for age, sex, and illness day. Other rows correspond to comparison for each illness phase, which included all values during that illness phase and were adjusted for age and sex. Analysis is based on linear regression with generalized estimating equations (variable of interest as outcome and plasma leakage as covariate). Effect (and 95% CI, *P* value) for grade 1 corresponds to mean difference in variable between grade 1 and 0. Effect (95% CI, *P* value) for grade 2 corresponds to mean difference in the variable between grade 2 and 0.

Abbreviations: CI, confidence interval; IQR, interquartile range; no. number of participants; No., number of measurements; RHI, reactive hyperemic index.

### Prognostic Potential of Endothelial Function on Days 1–3 for Developing Plasma Leakage in the Critical Phase

Although on illness days 1–3, the levels of arginase-1 tended to be higher and the l-arginine-to-ADMA ratio and RHI tended to be lower in patients who subsequently developed plasma leakage in the critical phase (days 4–6), these parameters were not found to be prognostic using logistic regression and the predefined cutoff of *P* < .01 (Supplementary Table 3*A*).

### Correlation of Endothelial Function and Plasma l-Arginine, Asymmetric Dimethylarginine, and Arginase-1

There was a negative correlation between RHI and plasma arginase-1 (ρ = –0.21; *P* = .001) and a positive correlation with l-arginine levels (ρ = 0.27; *P* = .001). Levels of arginase-1 had a strong negative correlation with l-arginine (ρ = –0.48; *P* < .001) and borderline correlation with ADMA (ρ = –0.19; *P* = .010). l-arginine was positively correlated with ADMA (ρ = 0.40; *P* < .001). We found no correlation between levels of arginase-1 and absolute neutrophil count or monocyte count, nor was there any correlation between enrollment arginase-1 and liver enzyme (aminotransferase) levels. Taken together, these results indicate a strong relationship between endothelial function/NO bioavailability with low levels of serum l-arginine and high levels of arginase.

## DISCUSSION

We have shown that endothelium-dependent vasodilation is impaired in dengue and also in OFIs during the acute phase of illness. Contradicting our first hypothesis, we found no difference between endothelial function or l-arginine, arginase-1, and ADMA levels between dengue and OFIs. However, in agreement with our second hypothesis, among patients with confirmed dengue we found that worse endothelial dysfunction was associated with more severe plasma leakage, apparent already in the early febrile phase. Although there was no association with the levels of l-arginine and arginase-1 between the plasma leakage grades, lower l-arginine and higher arginase levels occurred in the early febrile phase of illness and correlated with RHI, suggesting they are involved in the endothelial dysfunction and low NO bioavailability observed in dengue infections. This differs from other endothelial biomarkers such as angiopoietin-2 and VCAM-1 that tend to peak during the critical phase and suggests that hypoargininemia, high arginase, and reduced NO bioavailability are some of the earliest abnormalities in the pathophysiology of dengue [12].

Our results differ from the only other study assessing endothelial function in dengue, performed in Singaporean adults, which demonstrated higher RHI indices in patients with dengue hemorrhagic fever (DHF) compared to uncomplicated dengue [18]. The differences may be due to one or a combination of the following: the Singaporean patients were all older with milder disease, with only 6 DHF patients being hospitalized; the time of enrollment was later in the disease course, when we found a rebound increase in the RHI; and finally the old DHF classification may not be comparable to the severity grades used in our study.

We found that endothelial function was also impaired in the OFI group (which were predominantly other viral illnesses) in the early febrile phase compared with follow-up. The RHI was lower on days 1–3, suggesting that endothelial function and NO bioavailability is reduced early in many infectious diseases, potentially reflecting the influence of nonspecific inflammatory mediators on the vascular system, including proinflammatory cytokines such as tumor necrosis factor alpha (TNF-α) and interleukin 6 [19]. TNF-a can reduce endothelial NO through reduced availability of l-arginine and suppression of eNOS [20]. Further evidence for the role of TNF on endothelial function comes from therapeutics of anti-TNF monoclonal antibodies, which have demonstrated marked transient improvement in endothelial function during treatment [21]. However, although endothelial dysfunction appears to be a common phenomenon in many inflammatory and infectious conditions [22], the degree of impairment also appears to be related to disease severity. One study in adult sepsis demonstrated impaired endothelium-dependent vasodilation that was worse in patients with severe sepsis compared to those with moderate sepsis without organ failure [23]. Similarly, in malaria the degree of endothelial dysfunction has been found to associate with disease severity [24, 25].

A mechanism involving hypoargininemia and increased levels of arginase activity has been proposed in sepsis [26], and it is possible that similar derangements occur in many infectious diseases including malaria and dengue. The source of arginase-1 needs further research; possibilities include release from hepatocytes, but levels did not correlate with liver enzymes. Neutrophil-derived arginase-1 is also a possibility and although we did not demonstrate a correlation between arginase-1 and absolute neutrophil count, a study investigating the early whole blood transcriptional signature of dengue identified arginase-1 and several neutrophil-associated transcripts as being more abundant in dengue patients who progressed to shock [27]. Other possible mechanisms for hypoargininemia in dengue include poor nutritional intake during the acute illness, or leakage out of capillaries. However the levels of l-arginine were lowest in the febrile phase rather than the critical phase, when protein leakage is most marked in dengue. A more likely explanation is that high arginase-1 levels result from early activation and upregulation of neutrophil arginase-1 expression, along with release from neutrophil degranulation; l-arginine is consumed, causing depletion within endothelial cells and resulting in impaired NO bioavailability. We found no changes in levels of ADMA, making competitive inhibition of eNOS as a cause of the endothelial dysfunction less likely; however, the associated inflammatory response, specifically the inhibitory effect of TNF-α on eNOS, may play a role [28].

The timing of the endothelial dysfunction has potentially important implications for therapeutic interventions, particularly those that target the endothelium [29]. A recent trial of lovastatin in adult dengue given within 72 hours of fever did not have an impact on any clinical outcomes [30]. Future trials investigating endothelial modulating therapies should consider either administering the drug earlier (day 1) or targeting patients with evidence of early endothelial dysfunction.

The consequences of impaired endothelial NO release could have several important effects in dengue, not only by impairing vasomotion and altering microcirculatory tissue perfusion, but also may impact vascular permeability directly [12]. Both increased production as well as reduced endothelial NO release can influence microvascular permeability [31]. NO may protect vascular barrier function by inhibiting cellular adhesion. Low NO bioavailability can cause increased platelet and leukocyte adhesion to the vascular endothelial surface [32], and increased albumin leakage has been demonstrated in post capillary venules after NO inhibition [33]. Decreased NO levels also result in increased exocytosis of endothelial Weibel-Palade bodies and release of angiopoietin-2, which has been shown to cause glycocalyx degradation and increased vascular permeability [34, 35]. NO may also protect the microvasculature against mast cell degranulation products and associated permeability alterations [36, 37].

There were several limitations to our study. First, only a small number of patients with severe dengue were studied using EndoPAT, as many of the ICU study participants were excluded due to platelet counts <20 × 10^9^/L, and this may have underestimated the associations between severe dengue and endothelial dysfunction. The numbers of patients enrolled early in the febrile phase who subsequently developed shock were also small, making the prognostic models less robust. Last, plasma levels of arginase-1 may not reflect the activity of the enzyme, and arginase-1 activity assays should be used to confirm that high levels equate to increase enzymatic activity.

## CONCLUSIONS

This study has shown that impaired endothelial function is associated with dengue plasma leakage severity. Importantly, we found that endothelial dysfunction occurred early in the course of the disease, with worse function already apparent in the first 72 hours of fever in patients who subsequently developed severe plasma leakage. Endothelial dysfunction as a surrogate for NO bioavailability correlated with lower l-arginine and higher arginase-1 levels, but not ADMA, suggesting that hypoargininemia resulting from high arginase-1 levels plays a role. These findings provide important new information regarding the pathophysiology of progression to severe disease in dengue. Clinical endothelial function tests could be considered not only for screening patients for enrollment in therapeutic intervention trials, but potentially also as a surrogate endpoint for such trials.

## Supplementary Data

Supplementary materials are available at *Clinical Infectious Diseases* online. Consisting of data provided by the authors to benefit the reader, the posted materials are not copyedited and are the sole responsibility of the authors, so questions or comments should be addressed to the corresponding author.

## Supplementary Material

Supplementary_Data

## Notes

***Acknowledgments.*** We thank all the medical and nursing staff at the National Hospital for Tropical Diseases in Hanoi and the Hospital for Tropical Diseases in Ho Chi Minh City for patient recruitment and management; Kieu Nguyen Tan Thanh, Su Nguyen Thi Minh, and all the study nurses for their help coordinating the studies; and the Oxford University Clinical Research Unit dengue laboratory staff for sample processing.

***Financial support.*** This work was supported by the Wellcome Trust (grant number 100562/Z/12/Z) and the International Research Consortium on Dengue Risk Assessment, Management and Surveillance (IDAMS) (EU grant number FP7-281803; publication reference number IDAMS 47).

***Potential conflicts of interest.*** All authors: No reported conflicts of interest. All authors have submitted the ICMJE Form for Disclosure of Potential Conflicts of Interest. Conflicts that the editors consider relevant to the content of the manuscript have been disclosed.
