# Supplementary material for: Endothelial Nitric Oxide Pathways in the Pathophysiology of Dengue: A Prospective Observational Study
Source: Clin Infect Dis. 2017 Jun 29;65(9):1453–61. doi: 10.1093/cid/cix567 (PMC5850435; doi:10.1093/cid/cix567)
Supplement: Supplementary_Data [file cix567_suppl_supplementary-data.docx]

**Supplementary data**

**Table 1a. Association between ADMA levels and plasma leakage by illness phase in dengue patients**

|  |  |  | **No Plasma leakage (Grade 0)** |  |  | **Plasma leakage Grade 1** |  |  | **Plasma leakage Grade 2** |  | **Grade 1 vs 0** |  |  | **Grade 2 vs 0** |  |
| --- | --- | --- | --- | --- | --- | --- | --- | --- | --- | --- | --- | --- | --- | --- | --- |
|  | **n** | **N** |  | **n** | **N** |  | **n** | **N** |  | **Effect** | **95% CI** | **P** | **Effect** | **95% CI** | **P** |
| **ADMA ng/ml** | **23** | **45** | **0.5 (0.4, 0.6)** | **8** | **15** | **0.6 (0.5, 0.7)** | **19** | **37** | **0.6 (0.5, 0.7)** | **0.08** | **(-0.01, 0.17)** | **0.094** | **0.00** | **(-0.08, 0.09)** | **0.916** |
| Day 1-3 | 17 | 17 | 0.5 (0.4, 0.6) | 4 | 4 | 0.6 (0.5, 0.6) | 4 | 5 | 0.4 (0.4, 0.6) | 0.17 | (0.04, 0.30) | 0.010 | 0.08 | (-0.04, 0.19) | 0.188 |
| Day 4-6 | 20 | 20 | 0.5 (0.4, 0.6) | 7 | 7 | 0.6 (0.5, 0.6) | 11 | 12 | 0.5 (0.5, 0.7) | 0.05 | (-0.04, 0.15) | 0.269 | 0.03 | (-0.10, 0.15) | 0.676 |
| Day 7-13 | 8 | 8 | 0.5 (0.4, 0.6) | 3 | 4 | 0.6 (0.6, 0.8) | 15 | 20 | 0.6 (0.6, 0.7) | 0.19 | (-0.03, 0.42) | 0.091 | 0.05 | (-0.11, 0.21) | 0.529 |
| Day >13 | 11 | 11 | 0.6 (0.5, 0.6) | 5 | 5 | 0.7 (0.6, 0.8) | 15 | 16 | 0.6 (0.5, 0.7) | 0.18 | (0.05, 0.32) | 0.008 | 0.09 | (-0.03, 0.21) | 0.136 |

*Data are presented as medians (IQR). n corresponds to number of participants, N corresponds to number of measurements. For each variable, the first row corresponds to the overall comparison which included all values except values on day >13, and were adjusted for age, sex and illness day. Other rows correspond to comparison for each illness phase, which included all values during that illness phase and were adjusted for age and sex. Effect (and 95%CI, P) for grade 1 corresponds to mean difference in variable between grade 1 and 0. Effect (95%CI, P) for grade 2 corresponds to mean difference in the variable between grade 2 and 0.*

**Table 2a. Association between endothelial function, L-Arginine, Arginase and mucosal bleeding by illness phase**

|  |  |  | No bleeding |  |  | Bleeding |  |  |  |
| --- | --- | --- | --- | --- | --- | --- | --- | --- | --- |
| Characteristic | n | N |  | n | N |  | Effect | 95% CI | P |
| **Arginase**  **ng/ml** | **43** | **83** | **106.8 (63.7, 163.7)** | **11** | **22** | **134.8 (111.4, 184.4)** | **6.73** | **(-39.42, 52.89)** | **0.775** |
| Day 1-3 | 18 | 19 | 156.6 (99.6, 219.5) | 7 | 7 | 184.4 (127.0, 221.4) | 4.66 | (-76.30, 85.62) | 0.910 |
| Day 4-6 | 34 | 35 | 106.8 (65.3, 164.9) | 6 | 6 | 86.3 (69.7, 105.2) | -79.58 | (-163.60, 4.45) | 0.063 |
| Day 7-13 | 24 | 29 | 96.8 (47.2, 122.1) | 6 | 9 | 170.3 (133.2, 184.4) | 43.68 | (-6.80, 94.16) | 0.090 |
| Day >13 | 29 | 30 | 45.1 (25.4, 92.1) | 6 | 6 | 123.4 (78.7, 177.5) | 35.55 | (-21.41, 92.51) | 0.221 |
|  |  |  |  |  |  |  |  |  |  |
| **L-Arginine**  **ng/ml** | **43** | **83** | **54.6 (37.5, 70.5)** | **11** | **22** | **40.8 (33.8, 54.9)** | **-12.52** | **(-24.28, -0.75)** | **0.037** |
| Day 1-3 | 18 | 19 | 47.2 (41.1, 65.0) | 7 | 7 | 37.1 (35.2, 51.7) | -11.20 | (-29.20, 6.80) | 0.223 |
| Day 4-6 | 34 | 35 | 48.5 (33.8, 66.7) | 6 | 6 | 48.4 (37.0, 60.2) | -10.07 | (-32.34, 12.21) | 0.376 |
| Day 7-13 | 24 | 29 | 65.9 (48.2, 75.7) | 6 | 9 | 36.2 (29.0, 51.1) | -15.83 | (-31.76, 0.10) | 0.051 |
| Day >13 | 29 | 30 | 88.7 (74.8, 142.6) | 6 | 6 | 53.3 (45.0, 65.9) | -30.65 | (-47.85, -13.45) | 0.001 |
|  |  |  |  |  |  |  |  |  |  |
| **RHI** | **101** | **199** | **1.9 (1.5, 2.2)** | **57** | **109** | **1.8 (1.5, 2.2)** | **-0.07** | **(-0.21, 0.07)** | **0.335** |
| Day 1-3 | 52 | 53 | 1.6 (1.5, 2.1) | 24 | 25 | 1.5 (1.3, 1.8) | -0.14 | (-0.33, 0.05) | 0.160 |
| Day 4-6 | 77 | 82 | 1.9 (1.5, 2.3) | 35 | 37 | 1.8 (1.5, 2.1) | -0.01 | (-0.22, 0.21) | 0.942 |
| Day 7-13 | 59 | 64 | 2.0 (1.7, 2.4) | 39 | 47 | 2.0 (1.6, 2.5) | -0.07 | (-0.27, 0.14) | 0.526 |
| Day >13 | 53 | 54 | 1.7 (1.5, 2.1) | 23 | 23 | 2.0 (1.4, 2.2) | 0.02 | (-0.25, 0.30) | 0.880 |

*Data are presented as medians (IQR).All analyses were based on logistic regression with generalized estimation equation.* n corresponds to number of participants, N corresponds to number of measurements. *Top row (in bold) represents the overall comparison including measurements from days 1-13. The analysis was adjusted for age, sex, illness day at enrolment, and illness day of measurement. RHI- Reactive hyperaemic Index*

**Table 3a: Potential predictors on days 1-3 for developing plasma leakage during the critical phase**

|  | n | No plasma leakage (N=68) | n | Plasma leakage (N=18) | OR | (95% CI) | p value |
| --- | --- | --- | --- | --- | --- | --- | --- |
| Arginase | 17 | 136.3 (103.4, 221.2) | 7 | 195.2 (150.4, 248.7) | 1.00 | (0.98, 1.01) | 0.614 |
| L-Arginine | 17 | 47.2 (39.9, 62.3) | 7 | 47.6 (37.9, 53.1) | 1.05 | (0.97, 1.18) | 0.245 |
| L-Arginine:ADMA | 17 | 108.9 (79.8,118.2) | 7 | 89.6 (85.1,91.9) | 0.98 | (0.89,1.03) | 0.524 |
| RHI | 57 | 1.8 (1.5, 2.1) | 15 | 1.5 (1.3, 1.6) | 0.12 | (0.01, 0.80) | 0.028 |

*Data are presented as median (IQR). n corresponds to number of participants. Comparison for each parameter was based on logistic regression, and was adjusted for age, sex and hospitalization.*

**Figure 1a: Scatterplot of L-arginine levels in patients with dengue versus OFI by illness phase**


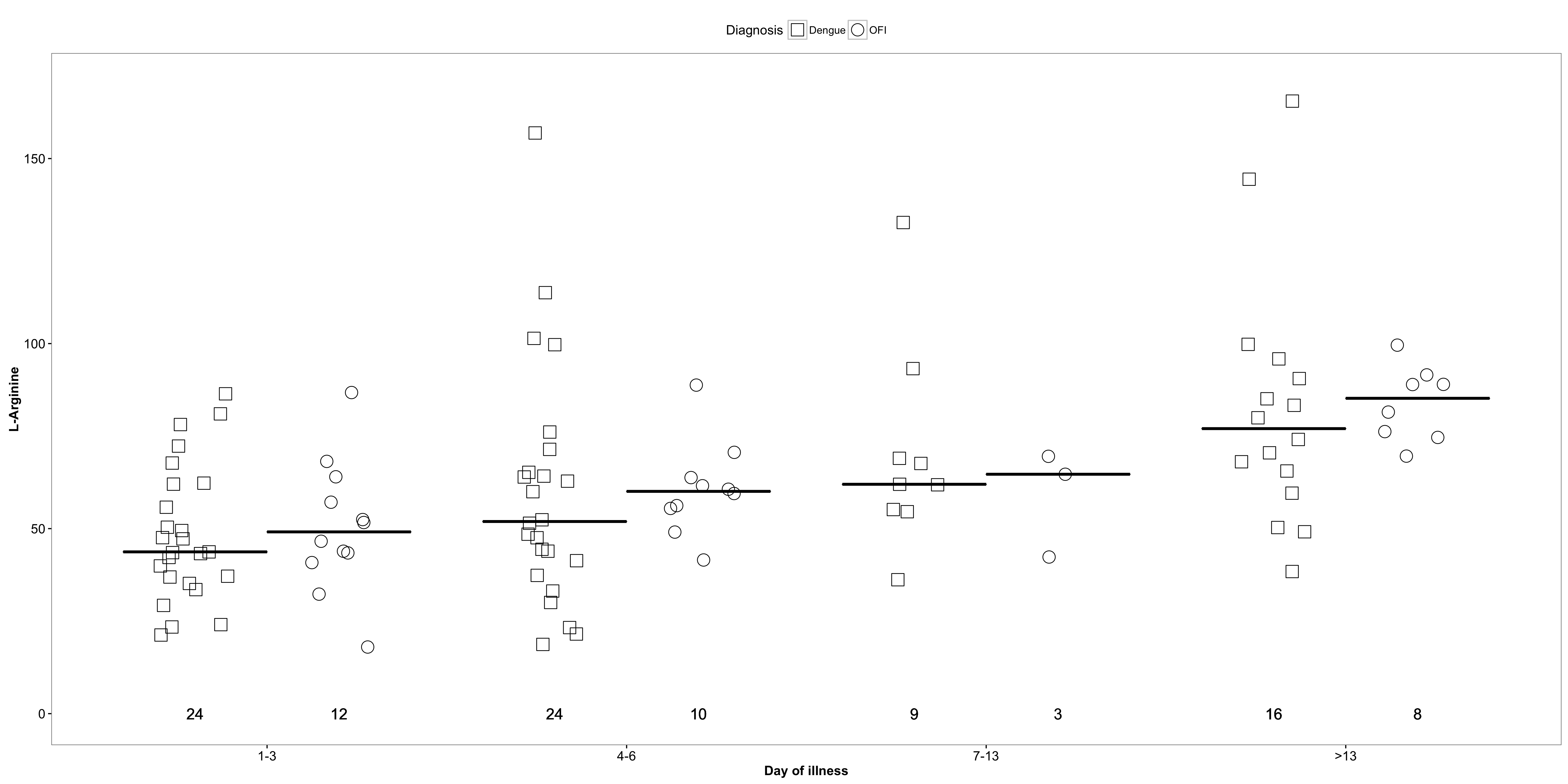


*Short grey line represents the median value of L-Arginine on each illness phase. The number at the bottom of the graph represents the number of patients that contributed to each group. This graph is based on 42 patients from the outpatient arm with at least 1 measurement of L-Arginine.*

**Figure 2a: Scatterplot of arginase-1 levels in patients with Dengue and OFI by illness phase**


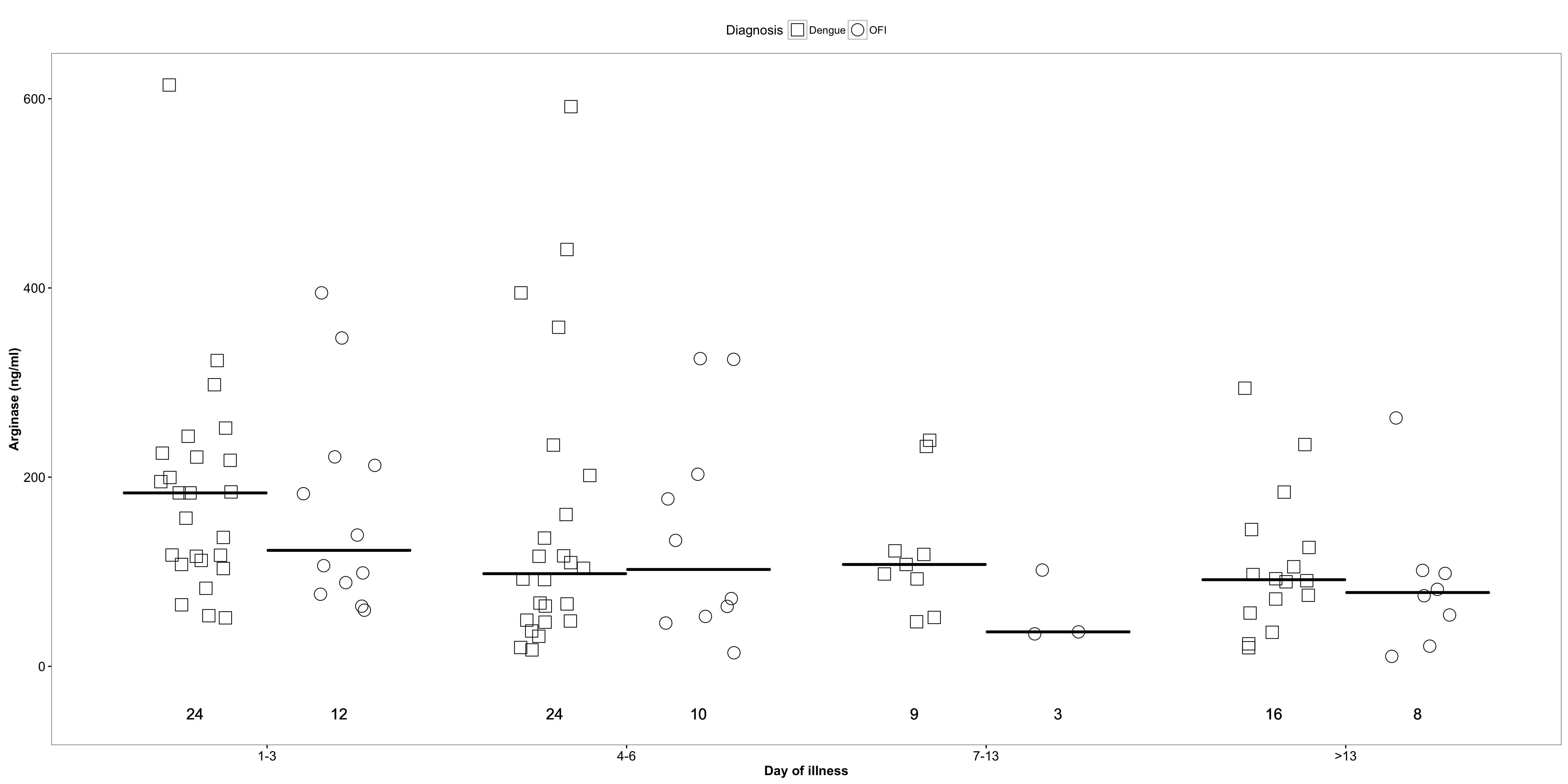


*Short grey line represents the median value of arginase at each illness phase. The number at the bottom of the graph represents the number of patients that contributed to each group. This graph is based on 42 patients with at least 1 measurement of arginase.*

**Figure 3a: Scatterplot of L-arginine levels in dengue patients by plasma leakage severity and illness phase**


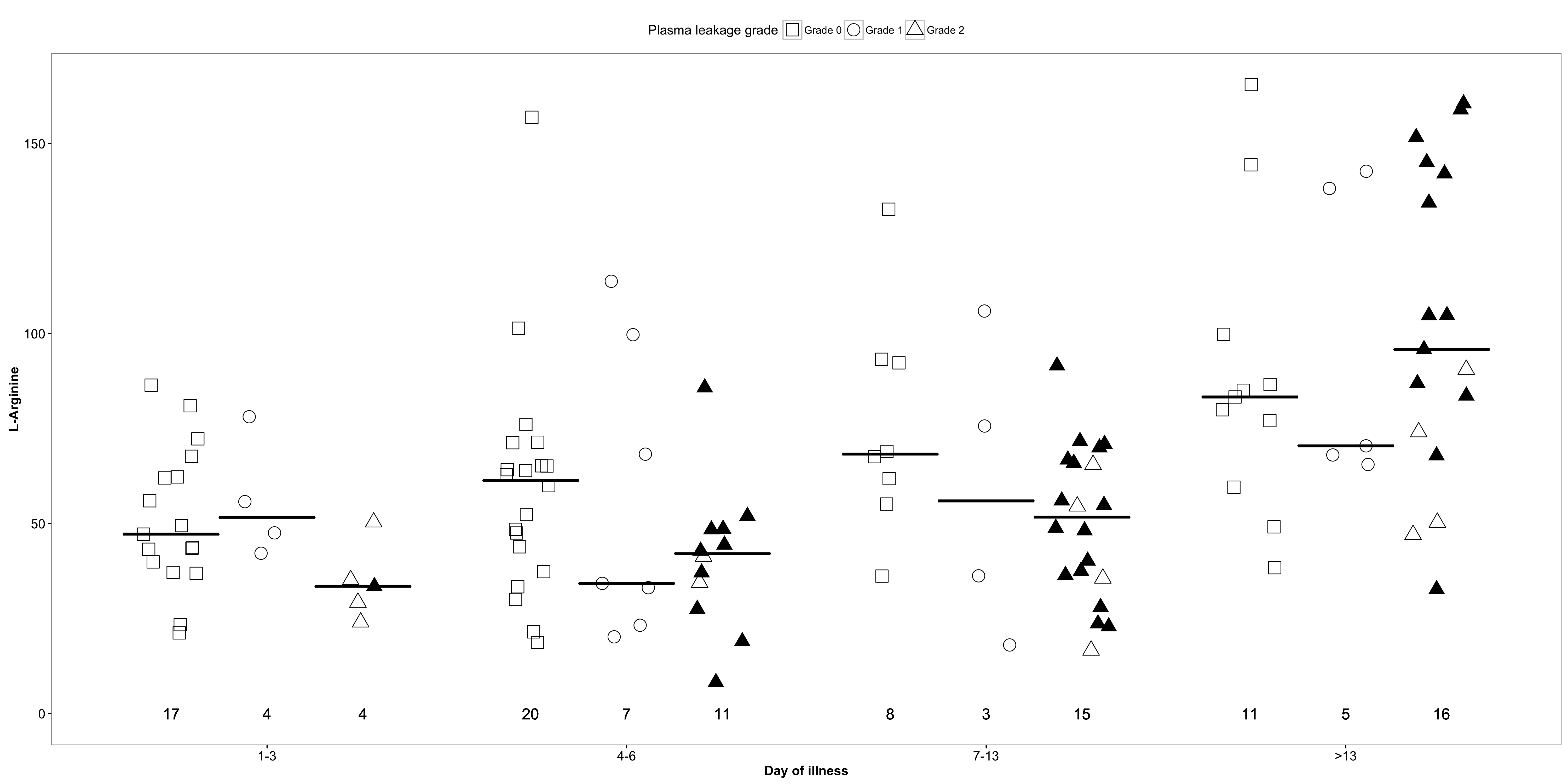


*Short grey line represents the median value of L-arginine ratio for each illness phase. The number represents the number of patients that contributed to each group. These graphs are based on 54 patients with at least 1 measurement.*
